# One-stage rotator cuff repair in stiff shoulders shows comparable range of motion, clinical outcome and retear rates to non-stiff shoulders: a systematic review

**DOI:** 10.1186/s13018-023-04104-w

**Published:** 2023-08-22

**Authors:** Lei Yao, Yinghao Li, Tao Li, Long Pang, Jian Li, Xin Tang

**Affiliations:** 1 Sports Medicine Center, West China Hospital, Sichuan University, Chengdu, China; 2 Department of Orthopedics and Orthopedic Research Institute, West China Hospital, Sichuan University, Chengdu, 610041 China

**Keywords:** Rotator cuff tear, Stiff shoulder, One-stage treatment, Arthroscopic repair, Retear

## Abstract

**Background:**

One-stage treatment involving rotator cuff repair and shoulder capsule release is mainly used to treat patients with rotator cuff tears (RCTs) and concomitant shoulder stiffness. Despite the increasing attention to the efficacy and safety of one-stage treatment, controversy still remains. Therefore, this systematic review aims to summarize the indications, operation procedure and rehabilitation protocol, and compare the range of motions (ROMs), functional outcomes and retear rates of one-stage treatment for RCTs in stiff shoulders and non-stiff shoulders.

**Methods:**

Multiple databases (PubMed, the Cochrane Library, Embase and MEDLINE) were searched for studies that investigated outcomes after one-stage treatment for RCTs concomitant with shoulder stiffness compared with rotator cuff repair for RCTs alone, according to the Preferred Reporting Items for Systematic Reviews and Meta-Analyses criteria. Descriptive statistics, including range of motion, patient-reported outcome and retear rate, are presented without meta-analysis due to the heterogeneity and low levels of evidence.

**Results:**

A total of 9 cohort studies were included, with 305 patients treated with one-stage treatment involving rotator cuff repair and simultaneous shoulder capsular release and 1059 patients treated with rotator cuff repair alone. Patients in both groups had significant symptom improvement and functional recovery after the one-stage treatment for the stiffness group and standard repair for the non-stiffness group, and most patients could return to normal life and work within 6 months after the operation. The retear rate in the one-stage treatment group was not higher than that in the rotator cuff repair group. No statistically significant differences between the two groups were observed in terms of range of motion and patient-reported outcomes in the vast majority of studies at the final follow-up, including the visual analog scale for pain, the Constant score, the American Shoulder and Elbow Surgeons score, the University of California Los Angeles Shoulder Score, the Oxford shoulder score and the Simple Shoulder Test.

**Conclusion:**

One-stage treatment for RCTs in stiff shoulders provides comparable ROM and patient-reported clinical outcomes as rotator cuff repair for non-stiff RCTs. In addition, the rate of postoperative retear in stiff shoulder treated with one-stage treatment was not higher than in non-stiff shoulders.

## Introduction

Shoulder stiffness and rotator cuff tears (RCTs) are two common shoulder disorders that affect joint function and quality of life [1]. Limited range of motion (ROM) and pain are the main manifestations of a stiff shoulder, and non-operative treatments, including oral medication, intra-articular injection and physical therapy, are commonly used [2]. Surgical intervention is needed for patients who do not respond to or tolerate conservative treatment. Shoulder capsular release, such as manipulation under anesthesia (MUA) and/or arthroscopic capsular release, are common options [3, 4]. Arthroscopic rotator cuff repair (ARCR) can lead to satisfactory functional outcomes in 80% of cases and is recommended as an optimal treatment for symptomatic RCTs [5, 6]. However, shoulder stiffness with concomitant RCTs remains a difficult problem for surgeons, and the optimal treatment remains controversial [7–10].

Several studies have confirmed that insufficient preoperative ROM is a risk factor for postoperative stiffness [7, 8], which may lead to a prolonged rehabilitation process and decreased satisfaction. Thus, some surgeons prefer to delay the operation until the recovery of ROM and relief of symptoms [2]. However, two-stage treatment involving conservative treatment prior to ARCR did not achieve more desirable outcomes than one-stage treatment [9, 10]. This strategy may aggravate RCTs due to a prolonged treatment period, reduce patient satisfaction and create an additional financial burden; furthermore, not all patients can tolerate pain during the rehabilitation process [9, 10]. Meanwhile, some surgeons believe that RCTs result in persistent pain and capsular contracture, which may exacerbate the stiffness of the shoulder [11–13]. Therefore, these surgeons suggest that RCTs combined with a stiff shoulder should be treated with a one-stage treatment consisting of ARCR with simultaneous shoulder capsular release. Several case series have confirmed the feasibility and good outcomes of one-stage treatment for stiff shoulder combined with RCTs [14, 15]. Reports on retear rates varied among studies, and while some studies have found no difference in retear rates, others have found that stiffness seems to have a protective effect that leads to a lower retear rate [1, 16, 17]. Thus, the differences in outcomes and retear rates between one-stage treatment and isolated ARCR for RCTs remain undefined.

Therefore, this systematic review aims to summarize the indications, operation procedure and rehabilitation protocol, and compare the ROMs, functional outcomes and retear rates of one-stage treatment for RCTs in stiff shoulders and non-stiff shoulders. We hypothesized that one-stage treatment for RCTs combined with shoulder stiffness can provide comparable outcomes compared to ARCR for RCTs alone.

## Materials and methods

### Literature search

The Preferred Reporting Items for Systematic Reviews and Meta-Analyses (PRISMA) criteria were followed to conduct this study [18]. Multiple databases, including PubMed, the Cochrane Library, Embase and MEDLINE, were searched from database inception to April 30, 2023, using the retrieval terms ((stiff shoulder OR frozen shoulder OR adhesive capsulitis) AND (rotator cuff)). The references of the included literature were screened for potential inclusion. This study has been registered with PROSPERO (CRD42022355490).

### Eligibility criteria

The criteria for the included studies were: (1) clear definition of stiff shoulder with restricted ROM under anesthesia similar to the Upper Extremity Committee of ISAKOS [2], (2) comparison of one-stage treatment for RCTs combined with stiff shoulder and ARCR for RCTs alone, (3) at least 1 year of follow-up and (4) studies written in English. The exclusion criteria were: (1) cadaveric or animal studies, (2) conference abstracts, reviews or book chapters and (3) non-peer-reviewed studies. The first two authors independently screened the titles of the retrieved studies and excluded irrelevant studies. Then, the titles and abstracts were screened according to the inclusion and exclusion criteria. Full-text reviewing was conducted for all eligible studies. Any disagreement was resolved by the participation of a senior shoulder arthroscopy surgeon in a three-person evaluation.

### Data extraction

The baseline data extracted included first author, publication year, sample size, sex, age, follow-up time, incidence of diabetes mellitus, definition of stiff shoulder, surgical technique and rehabilitation protocol. The outcomes included ROM at different stages, functional scores, pain scores and retear rates. ROM included forward flexion (FF), abduction, external rotation (ER) and internal rotation (IR). Functional scores included the Constant shoulder score, the American Shoulder and Elbow Surgeons (ASES) score, the University of California Los Angeles Shoulder Score (UCLA), the Oxford shoulder scores and the Simple Shoulder Test (SST). The visual analog scale (VAS) was used to assess patient-reported pain. All data were extracted by one author and checked by another.

### Risk of bias and quality assessment

The Risk of Bias in Non-randomized Studies of Interventions (ROBINS-I) and Methodological Index for Non-Randomized Studies (MINORS) tools were used to appraise the included studies’ risk of bias and quality [19, 20]. The ROBINS-I tool assesses studies on the basis of confounding, selection of participants, classification of interventions, deviations from intended interventions, missing data, measurement of outcomes and selection of reported results. The overall risk of bias for each study was judged as “low,” “moderate,” “serious” or “critical.” The MINORS tool represents a 12-item assessment of methodological value for comparative studies. The maximum possible score is 24 for the included study, with higher scores generally indicating higher methodological quality and lower risk of bias. Two authors independently scored the studies, and an interrater reliability was calculated using the Cohen kappa statistic.

### Statistical analysis

Low levels of evidence and study heterogeneity precluded meta-analysis, and all data were summarized descriptively according to previous recommendations [21]. Forest plots of proportions are presented without pooled weighted means using R Foundation for Statistical Computing (version 4.0.3; Vienna, Austria).

## Results

### Literature search

**Fig. 1:**
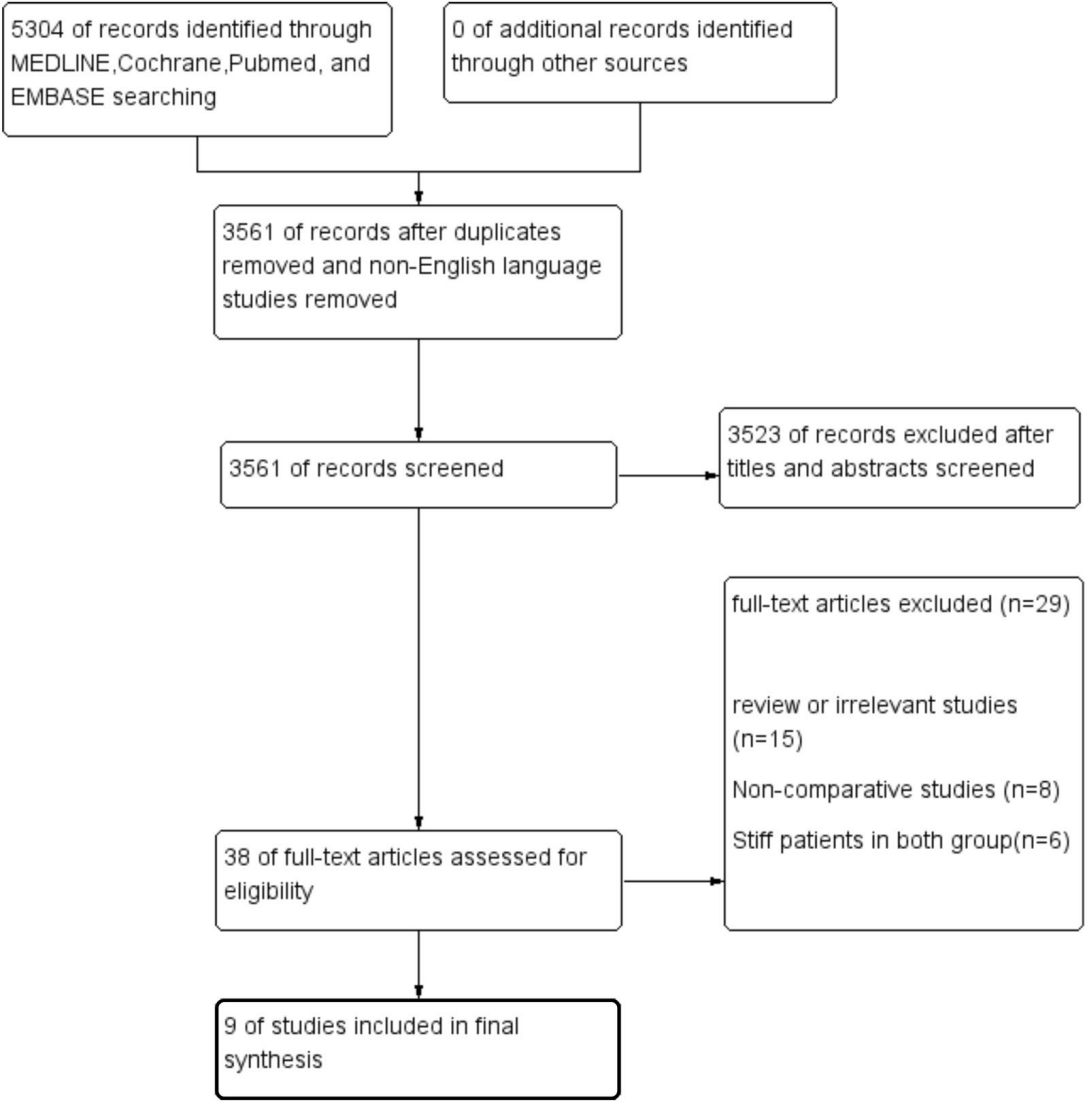
Preferred Reporting Items for Systematic Reviews and Meta-Analyses (PRISMA) study selection flowchart

A total of 5304 records were retrieved. After duplicate articles and non-English written articles were removed, the titles and abstracts of a total of 3561 articles were screened according to the inclusion and exclusion criteria. Thirty-eight relevant articles were included for full-text screening. No additional articles were identified from the reference search, and 9 case‒control studies, including 8 level III studies and one level II study, were included (Fig. 1) [1, 16, 17, 22–27].

### Study quality and patient demographics

All included studies were determined to present a low to moderate risk of bias, according to the ROBINS-I tool. The average MINORS score was 19.2 (range 17–21) for the included studies, with a kappa value of 0.86.

A total of 1364 RCTs patients (613 males and 751 females) were assessed, with 305 patients (131 males and 174 females) in the stiffness group and 1059 patients (482 males and 577 females) in the non-stiffness group. The overall mean ages were 59.6 and 59.2 in the stiffness group and non-stiffness group, respectively. All studies had at least a 24-month mean follow-up period, ranging from 24 to 36.9 months, except the study by Oh et al. [16], which had a mean follow-up of 15.1 months. The prevalence of diabetes mellitus was reported in 8 studies, with 25.7% (range, 10–34.5%) in the stiffness group and 12.5% (range, 1.1–19.5%) in the non-stiffness group [1, 16, 17, 22–25, 27]. Furthermore, except for the study by Oh et al. [16], the prevalence of diabetes in the stiffness group was greater than 20.5% in the remaining seven studies [1, 17, 22–25, 27]. The seven studies that included only full-thickness tears all reported tear size according to the classification of DeOrio and Cofield [1, 16, 17, 22–25], and two others included partial-thickness tears [26, 27], one of which did not report the full-thickness tear size. Among them, four studies did not include massive rotator cuff tears, and the vast majority of patients had medium size tears, with 59.4% in the stiffness group and 61.5% in the non-stiffness group. Details for each study are provided in Table 1.

**Table 1:** Characteristics of Included Studies

Author	Journal	LOE	MINORS	ROBINS-1	Number Of Patients		Mean age, Yrs ()		Gender (M/F)		Mean Follow-up, mo		% Diabetes mellitus		DeOrio and Cofield classification of RCT partial/small/medium/large/massive	
					S	NS	S	NS	S	NS	S	NS	S	NS	S	NS
Cho [23]	AJSM	III	18	Moderate	15	30	59.8	56.1	13/2	18/12	29.8	32.4	33.3%	1.1%	0/2/11/2/0	0/4/20/6/0
[22]	Arthroscopy	III	20	Low	26	26	58.4	59.2	9/17	8/18	36.9	33.3	23.1%	15.4%	0/11/9/5/1	0/12/8/5/1
Ho [24]	Arthroscopy	III	21	Low	41	163	52.8	55.1	15/26	62/101	29.3	30.4	31.8%	14.1%	0/8/27/6/0	0/29/114/20/0
Jeong [1]	OJSM	III	19	Moderate	58	154	61.4	61.9	22/36	56/98	32.51	30.1	34.5%	19.5%	0/8/45/5*	0/22/104/28*
Kim [25]	AJSM	III	18	Moderate	39	320	58.1	58.0	21/18	166/154	34.8	32.6	20.5%	12.8%	0/7/30/2/0	0/62/205/42/11
Mak [26]	J ORTHOP	III	18	Moderate	25	22	56.9	58.9	13/12	12/10	> 24	> 24	NR	NR	3/13/7/1/1	4/8/8/0/1
McGrath [27]	JSES	III	17	Moderate	25	170	57.9	60.1	12/13	81/89	31.2	27.6	28%	3.5%	18/7**	75/95**
Oh [16]	Arthroscopy	III	21	Low	30	97	60.9	58.8	15/15	44/53	15.1	15.1	10%	10.3%	0/5/10/10/5	0/19/40/20/18
Zhang [36]	Arthroscopy	II	21	Low	46	77	66.7	66.7	11/35	35/42	24	24	21.7%	19.5%	0/46***	0/77***

LOE, level of evidence; MINORS, Methodological Index for Non-Randomized Studies; ROBINS-1, Risk of Bias in Non-randomized Studies of Interventions S, stiffness group; NS, non-stiffness group; AJSM, *the American Journal of Sports Medicine*; OJSM, *the Orthopaedic Journal of Sports Medicine*; J ORTHOP, *Journal of Orthopaedics*; JSES, *Journal of Shoulder and Elbow Surgery*; NR, not recorded

*Data were presented as RCT size of partial/small/medium/large and massive

**Data were presented as RCT size of partial/full thickness

***Data were presented as RCT size of partial/small and medium

### Shoulder stiff definition

All studies described the definition of a stiff shoulder based on the ROM examined under anesthesia (Table 2). FF ranging from 90° to 135° was considered a threshold for a stiff shoulder in all studies [1, 16, 17, 22–27]. ER at the side ranging from 20° to 40° was used in 6 studies [1, 16, 22, 23, 25, 27]. ER at abduction of 60° or 90° was used in 2 studies[24, 26]. IR relative to the vertebral level less than L3 or T12 was used in 5 studies [1, 16, 23, 25, 27]. Abduction less than 90° was used in one study [27].

**Table 2:** Shoulder Stiff Definitions, Surgical Technique and Rehabilitation Protocol of Included Studies

Author	Criteria for stiffness	Position	Operation for RCT	Operation for stiffness	Combined procedures	Rehabilitation Protocol
Cho [23]	FF < 120°, ER < 40°	Beach chair	Arthroscopic, single or double row with suture anchors	MUA: FF, ER, ER-90 IR-90, abduction	SAD	Sling immobilized, passive ROM, pendulum exercise immediately postoperatively Active ROM, strengthening exercise at 6 week postoperatively Return to work at 6-month postoperatively
Cho [22]	FF < 120° or ER < 30° or IR < L3	Lateral decubitus	Arthroscopic or Mini-open, double row with suture anchors	MUA: NR Release: rotator interval and anterior, inferior and posterior capsules	SAD	Abduction bracing, passive ROM, pendulum exercise immediately postoperatively Active ROM at 6 week postoperatively Strengthening exercise at 3-month postoperatively Return to work at 6-month postoperatively
Ho [24]	FF < 135°, ER-90 < 60°	Lateral decubitus	Arthroscopic, suture anchors	MUA: FF, ER-90, IR-90, abduction Release: anterior, inferior and posterior capsules	SAD, acromioplasty	Sling immobilized, passive ROM, active-assisted exercise immediately postoperatively Active ROM, strengthening exercise at 6 week postoperatively Return to work at 3–6 month postoperatively
Jeong [1]	FF < 120°, ER < 30°, IR < L3 or limited ROM visually identified	Lateral decubitus	Arthroscopic, double row with suture anchors	MUA: FF Release: rotator interval and anterior, inferior and posterior capsules	SAD, acromioplasty	Abduction bracing immediately postoperatively for 4–6 week Passive ROM at 4 week postoperatively Active-assisted ROM at full passive ROM recovered Strengthening exercise at 10–12 week postoperatively
Kim [25]	FF < 120° or ER < 30° or IR < L3	Lateral decubitus	Arthroscopic, single or double row with suture anchors	Release: rotator interval, anterior, inferior and posterior capsules, MGHL	Acromioplasty	Abduction bracing, active elbow–wrist ROM immediately postoperatively Passive ROM at 2–4 week postoperatively Active ROM at 4–12 week postoperatively Strengthening exercise at 12 week postoperatively
Mak [26]	FF < 135°, ER-90 < 90°	Beach chair	Arthroscopic, suture anchors	Release: rotator interval, anterior and inferior capsules, MGHL	SAD, acromioplasty	Sling immobilized, passive ROM, active-assisted exercise immediately postoperatively Active ROM, strengthening exercise at 6 week postoperatively Return to work at 3–6 month postoperatively
McGrath [27]	FF < 90°, ER < 20°, IR < T12, abduction < 90°	Beach chair	Arthroscopic, single row with suture anchors	MUA: FF, ER-90, IR-90, abduction Release: anterior, inferior and posterior capsules	Acromioplasty	Abduction bracing, active hand–elbow activity, pendulum exercise immediately postoperatively Passive ROM at 1 week for stiffness group instead of 2 week postoperatively Active ROM at 6 week postoperatively Strengthening exercise at 3-month postoperatively
Oh [16]	FF < 120° or ER < 30° or IR < L3	Lateral decubitus	Arthroscopic or Mini-open	MUA: FF, ER, ER-90, IR-90, extension, abduction Release: rotator interval, anterior and inferior capsules, MGHL	Acromioplasty, Distal clavicle resection for acromioclavicular arthritis, Debridement or repair for SLAP lesion	Abduction bracing, passive ROM immediately postoperatively Active ROM at 4–7 week postoperatively Strengthening exercise at 9–12 week postoperatively Return to work at 6-month postoperatively
Zhang [36]	FF < 100°	Beach chair	Arthroscopic, single or double row with suture anchors	MUA: FF, ER, ER-90, IR-90, extension, abduction	Acromioplasty	Sling immobilized immediately postoperatively Passive ROM, pendulum exercise at 2 week postoperatively Active ROM, strengthening exercise at 6 week postoperatively Return to work at 3-month postoperatively

FF, forward flexion; ER, external rotation at side; MUA, manipulation under anesthesia; ER-90, external rotation at 90°of abduction; IR-90, internal rotation at 90°of abduction; SAD, subacromial decompression; ROM, range of motion; IR, internal rotation; NR, not recorded; MGHL, middle glenohumeral ligament

### Operation procedure and rehabilitation protocol

Study-specific interventions are listed in Table 2. All patients underwent standard rotator cuff repair, and seven studies all performed arthroscopic surgery, while two studies performed arthroscopic or mini-open surgery. The percentage of all included patients who underwent arthroscopic repair was 91.8% in the stiffness group and 93.8% in the non-stiffness group. Patient positioning was described in all studies, with 4 studies using beach chair and 5 studies using lateral decubitus. Except for the study by Oh et al., which did not report the suture method, the remaining eight studies used the suture anchor technique to repair RCTs. McGrath et al. used single-row repair alone, while Cho et al. and Jeong et al. used double-row repair alone, and the other five studies performed single- or double-row repair based on tear size. Partial-thickness RCTs were converted to full-thickness RCTs before repair. In addition, subacromial bursectomy was also performed in five studies, and acromioplasty was performed in seven studies for all included patients. Oh et al. performed distal clavicle resection for acromioclavicular arthritis and debridement or repair for SLAP lesions.

Operations for stiffness included MUA and capsular release, with the MUA technique alone in 2 studies, the release technique alone in 2 studies and MUA combined with release in 5 studies. Six studies described the details of MUA, with 6 studies including mobilization in FF; 5 studies, ER-90, IR-90 and abduction; 2 studies, ER at side; and 1 study, extension. All MUAs were performed gently by the surgeon, with popping sound as the end point of the operation. Capsular release details were described in seven studies, with 7 studies performing release of anterior and inferior capsular; 5 studies, the rotator interval and posterior capsular; and 2 studies, the middle glenohumeral ligament.

All patients included underwent a progressive rehabilitation program. Accelerated postoperative rehabilitation protocols were used in eight included studies except Jeong et al. Abduction braces or slings were used in all patients immediately postoperatively for 4–7 weeks based on different studies and tear sizes, and passive ROM could be carried out during this time. Adjunct therapies during this stage included pendulum exercises, and active elbow, wrist and hand ROM. Active ROM was initiated after the brace was removed and strengthening exercise was started 6–12 weeks after surgery. Return to activity took place at 3–6 months.

### Clinical outcomes

#### Range of motion

Preoperative and postoperative ROM was analyzed in four dimensions: FF, ER, IR and abduction, with FF reported in 9 studies, ER and IR in 8 studies and abduction in 5 studies (Tables 3 and 4). All studies except Zhang et al. reported at least two dimensions. All studies reported significant improvements in FF. The mean FF improvement in the stiffness group ranged from 38° to 67.9°. There was no significant difference in mean FF between the two groups at the last follow-up in any of the nine studies. The mean postoperative FF in the stiffness group ranged from 121.1° to 175° and in the non-stiffness group, from 124.9° to 175°. None of the eight studies showed significant differences in ER at the last follow-up, and 7 studies reported ER at side except for the ER score of the constant score used by Mak et al. The mean improvement in the stiff group ranged from 13.9° to 42°. The postoperative ER ranged from 47.8° to 66.2° in the stiffness group and from 49.1° to 71° in the non-stiffness group. Of the eight studies that reported IR, only McGrath et al. showed a difference between the two groups at the last follow-up. The mean difference in IR relative to vertebral level between groups decreased from 6 vertebral segments preoperatively to 2 vertebral segments postoperatively. The remaining studies reported mean differences in IR between groups of less than one vertebral segment. The mean improvement of IR in the stiffness group was 5 to 7 vertebral segments. Five studies included abduction and reported significant improvements. The stiffness group had an average abduction improvement of 50° -72.4°. No significant difference was observed in mean abduction reported in 5 studies between the two groups at the last follow-up. The mean postoperative abduction in the stiffness group ranged from 113.5° to 173.7° and in the non-stiffness group, from 112.7° to 174.2°.

**Table 3:** Preoperative Range of Motion and Patient-Reported Outcomes of Included Studies

Author		FF (°)	ER (°)	IR	Abduction (°)	VAS Pain Score	Constant score	ASES Score	UCLA Score	Oxford Shoulder Score	SST score
Cho [23]	S	118.3	34.6	L4	112.5	6.5	44.6	NR	14.6	NR	2.7
	NS	163.4	55.0	T9	161.6	5.7	64.2	NR	17.9	NR	4.3
Cho [22]	S	100.6	24.2	L4.2	NR	6.6	NR	34.6	12.3	NR	NR
	NS	156.9	45.4	L1.7	NR	6.9	NR	39.9	15.7	NR	NR
Ho [24]	S	124	30	6*	120	8	45	39	14	NR	NR
	NS	175	60	30*	165	7	45	41	13	NR	NR
Jeong [1]	S	113.4	29.1	L0.5	73.9	4.6	31.6	35.5	NR	NR	NR
	NS	144.7	45.1	T8.5	114.5	4.9	53.4	48.7	NR	NR	NR
Kim [25]	S	95.9	17.4	L4.9	NR	NR	NR	NR	18.7	NR	NR
	NS	147.7	51.6	L2.4	NR	NR	NR	NR	22.5	NR	NR
Mak [26]	S	74.8	1.1**	2.3**	57.0	7.5	23.5	NR	12.1	40.1	NR
	NS	100.2	6.1**	6.8**	84.2	6.5	43.2	NR	14.8	32.3	NR
McGrath [27]	S	104	29	S2	81	NR	NR	NR	NR	NR	NR
	NS	150	55	L1	128	NR	NR	NR	NR	NR	NR
Oh [16]	S	128	37	L0.9	NR	6.3	44.4	37.3	NR	NR	1.5
	NS	163	69	T8.7	NR	5.9	60.0	49.6	NR	NR	3.6
Zhang [36]	S	66.8	NR	NR	NR	7.35	24.4	NR	NR	39.3	NR
	NS	117.4	NR	NR	NR	5.69	51.0	NR	NR	28.1	NR

FF, forward flexion; ER, external rotation at side; IR, internal rotation; VAS, visual analog scale; ASES Score, the American Shoulder and Elbow Surgeons score; UCLA score, the University of California Los Angeles Shoulder score; SST score, the Simple Shoulder Test score; S, stiffness group; NS, non-stiffness group; NR, not reported

*Data were presented as internal rotation in 90° of abduction

**Data were presented as component of Constant and Murley Shoulder Score

**Table 4:** Postoperative Range of Motion and Patient-Reported Outcomes of Included Studies

Author		FF (°)	ER (°)	IR	Abduction (°)	VAS Pain Score	Constant score	ASES Score	UCLA Score	Oxford Shoulder Score	SST score
Cho [23]	S	166.7	48.5	T9	173.7	0.8	94.3	NR	33.2	NR	11.3
	NS	170.2	53.4	T8	174.2	0.8	92.6	NR	33.2	NR	11.3
Cho [22]	S	168.5	66.2	T9.9	NR	1.4	NR	87.7	33.0	NR	NR
	NS	169.2	68.8	T10.3	NR	1.7	NR	85.2	32.6	NR	NR
Ho [24]	S	175	60	33*	170	1.5	92	90	33	NR	NR
	NS	175	60	30*	170	1.3	90	88	32	NR	NR
Jeong [1]	S	154.1	47.8	T7.52	146.3	1.0	61.1	80.7	NR	NR	NR
	NS	155.4	49.1	T7.51	140.2	1.1	72.3	86.3	NR	NR	NR
Kim [25]	S	#	#	#	NR	NR	NR	NR	#	NR	NR
	NS	#	#	#	NR	NR	NR	NR	#	NR	NR
Mak [26]	S	121.1	8.8**	7.4**	113.5	1.1	70.4	NR	28.3	15.3	NR
	NS	126.5	9.0**	6.6**	112.7	1.7	69.2	NR	28.8	16.6	NR
McGrath [27]	S	161	59	T12	148	NR	NR	NR	NR	NR	NR
	NS	166	59	T10	150	NR	NR	NR	NR	NR	NR
Oh [16]	S	166	64	T9.3	NR	1.7	79.1	86.6	NR	NR	9.2
	NS	169	71	T8.4	NR	1.9	81.7	83.9	NR	NR	8
Zhang [36]	S	130.6	NR	NR	NR	1.0	68.6	NR	NR	15.9	NR
	NS	124.9	NR	NR	NR	0.9	69.6	NR	NR	16.3	NR

FF, forward flexion; ER, external rotation at side; IR, internal rotation; VAS, visual analog scale; ASES Score, the American Shoulder and Elbow Surgeons score; UCLA score, the University of California Los Angeles Shoulder score; SST score, the Simple Shoulder Test score; S, stiffness group; NS, non-stiffness group; NR, not reported

*Data were presented as internal rotation in 90° of abduction

**Data were presented as component of Constant and Murley Shoulder Score

^#^Data could not be obtained from the original study, but there was no significant difference between the two groups

### Patient-reported outcomes

All studies used at least one patient-reported outcome to measure postoperative patient shoulder function. The preoperative and last follow-up results of VAS pain score, Constant score, ASES score, UCLA score, Oxford shoulder score and SST score are listed in Tables 3 and 4. According to previous studies, the MCID for RCRs of each outcome was 1.5 points for VAS, 10.4 points for Constant score, 21 points for ASES score, 6 points for UCLA score, 3.3 points for Oxford Shoulder Score and 4.3 points for SST [28–30].

All studies reported significant improvements based on baseline pain levels. The seven studies that used VAS pain scores had mean postoperative scores ranging from 0.8 to 1.9. The mean improvement in VAS was 3.6 to 6.5 points in the stiffness group and 3.8 to 5.7 points in the non-stiffness group, and all studies reached clinical significance using the MCID for VAS score.

Six studies reported Constant scores ranging from 61.1 to 94.3, with mean Constant score improvement ranging from 29.5 to 49.7 in the stiffness group and 18.6 to 45 in the non-stiffness group. All study improvements in Constant scores reached statistical significance and clinical significance based on a MCID of 10.4.

Four studies reported ASES scores ranging from 80.7 to 90, with mean ASES score improvement ranging from 45.2 to 53.1 in the stiffness group and 34.3 to 47 in the non-stiffness group. All study improvements in ASES scores reached statistical significance and clinical significance based on a MCID of 21.

Five studies reported postoperative UCLA scores ranging from 28.3 to 33.2, with mean UCLA score improvement ranging from 16.2 to 20.7 in the stiffness group and 14 to 19 in the non-stiffness group. All study improvements in UCLA scores reached statistical significance and clinical significance based on a MCID of 6.

Other outcomes included the Oxford Shoulder Score in 2 studies and the Simple Shoulder.

Test in 2 studies, with all showing improvement of statistically significant and clinically significant improvements based on MCIDs of 3.3 and 4.3, respectively.

### Retear rates

**Fig. 2:**
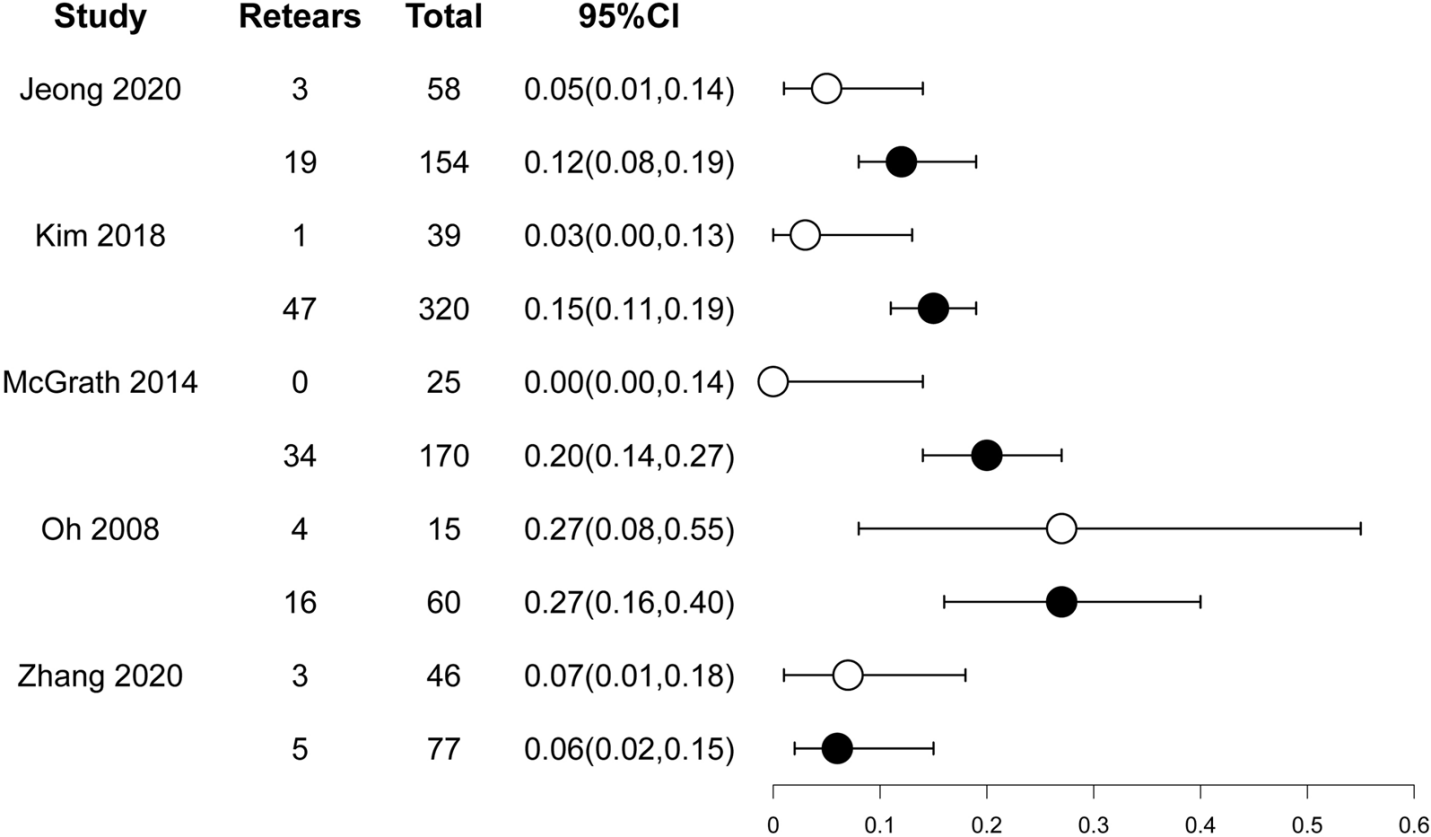
Forest plot for retear rates of the stiffness group versus the non-stiffness group. Circles indicate the retear rate, and error bars represent 95% confidence intervals (open circle, non-stiffness group; filled circle, stiffness group)

Rotator cuff integrity was evaluated in five studies using ultrasound or MRI postoperatively, both of which were thought to be highly accurate at detecting full-thickness tears in the postoperative setting, [31] with 183 patients in the stiffness group and 781 patients in the non-stiffness group. A retear was defined as Sugaya type IV and V tears in two studies using MRI [1, 25]. The other two studies using ultrasonography diagnosis defined retear as a local area of decreased echogenicity [17, 27]. Oh et al. used ultrasonography or computed tomography arthrography to evaluate postoperative retear, but the specific diagnostic criteria were not described [16]. Additionally, their study did not report why only 15/30 (50%) patients in the stiffness group and 60/97 (61.9%) patients in the non-stiffness group were selected to evaluate postoperative rotator cuff integrity, and 27% of patients in both groups were evaluated to have retears with no statistically significant. The remaining four studies that evaluated all patients showed postoperative retear rates ranging from 0 to 20%, with three finding a statistically lower retear rate (range, 0% to 5%) in the stiffness group than in the non-stiffness group (range, 12% to 20%) and one finding no difference between the two groups (stiffness group 7% vs non-stiffness group 6%) (Fig. 2).

## Discussion

The main finding of this systematic review was that one-stage treatment for RCTs in stiff shoulders provides comparable ROM and patient-reported clinical outcomes as RCR for non-stiff RCTs. In addition, the rate of postoperative retear in stiff shoulder treated with one-stage treatment was not higher than in non-stiff shoulders.

According to the Upper Extremity Committee of ISAKOS, stiff shoulder describes a patient who presents with restricted ROM [2]. Stiffness was the most common complication after ARCR, ranging from 4.9 to 32.7% [12, 32, 33]. Studies have confirmed that preoperative stiffness is a risk factor for postoperative stiffness [7, 8, 34]. Thus, one-stage treatment involving ARCR and shoulder capsular release has been reported for patients with stiff shoulders and RCTs. Sabzevari et al. [35] included four retrospective comparative studies for qualitative analysis. They demonstrated that one-stage treatment for non-massive RCTs concomitant with stiff shoulder may have comparable results to the treatment of isolated RCTs. Zhang et al. [36] included 17 articles and demonstrated that one-stage treatment can effectively treat stiff shoulder with RCTs. Furthermore, the similar retear rates compared with patients undergoing ARCR alone certified the safety of one-stage treatment. This systematic review involving more high-level studies showed that the ROM and patient-reported outcomes of the one-stage treatment group did not differ from those of the control group at the final follow-up. None of the results reached the MCID, which means that comparable results could be achieved with one-stage treatment at the final follow-up.

The vast majority of ARCR patients could return to work 6 months postoperatively using an accelerated postoperative rehabilitation protocol [37]. Previous studies have reported that patients with stiff shoulders required longer rehabilitation after ARCR [8, 34]. However, reports on the recovery patterns of one-stage treatment vary. The time point at which differences in the FF between groups disappeared was reported to be 6–12 months after the operation, whereas the time point for ER was reported to be 3–12 months after the operation in different studies [16, 22, 23]. Jeong et al. [1] found statistically significant differences in FF and ER, even 12 months postoperatively. The possible reason is that the study adopted a conservative rehabilitation strategy, with passive ROM starting in the fourth week postoperatively. Although the progression of ROM recovery varied in each study, the functional score and VAS score for pain at 6 months postoperatively showed similar results [1, 16, 22]. Most patients could return to full activity 6 months after surgery. Previous studies of one-stage treatment have also shown no less effectiveness than two-stage treatment for patients with 3 to 6 months of rehabilitation preoperatively [9, 10]. Chen et al. [38] reported that patients with symptoms lasting longer than 6 months were less likely to benefit from one-stage treatment than patients with symptoms lasting less than 6 months. Based on these studies, considering that most patients achieved satisfactory recovery of function within 6 months, one-stage treatment could be considered as soon as possible for patients with stiff shoulder combined with RCTs.

Retear after ARCR is a common and unwanted complication and one of the main reasons for reduced patient satisfaction, with reported rates ranging from 11 to 94% [39, 40]. Previous studies reported retear rates for one-stage treatment ranging between 6.1 and 13.4% [9, 38, 41]. However, few studies have compared the retear rate between one-stage treatment and ARCR. Oh et al. [16] and Zhang et al. [17] found no statistically significant difference between groups, while several studies found that one-stage treatment could provide better rotator cuff integrity protection [1, 25, 27]. Several reasons could have contributed to this outcome. First, preoperative stiffness may lead to changes in the pathological process of RCTs, resulting in more aggressive healing of the rotator cuff [8, 24]. Second, MUA and/or capsular release may reduce the tension of the repaired rotator cuff [42, 43]. Third, symptom duration may also influence the outcome [44]. Symptom duration was reported in three of five included studies, with a relatively shorter duration in the stiff group, and the results showed differences in retear rates in 2 groups [25, 27] and no difference in one group [16]. A longer symptom duration may lead to increased fatty infiltration and muscle atrophy [11, 42], which are risk factors for retear after operation [44]. In addition, confounding factors that we have not been able to assess may also affect the retear rate, such as tear size and rehabilitation protocol [45–47]. Further exploration should focus on the risk factors associated with retears after surgery for patients with stiff shoulders and RCTs.

The surgical release method may be a cofounding factor in this study. Due to the paucity of research, the most effective approach remains undefined. Chuang et al. [48] found that MUA combined with capsular release provided better ROM in FF and ER than MUA alone for patients with a stiff shoulder and RCTs. Park et al. [41] found no difference between groups, but MUA combined with capsular release may result in better ER and functional outcomes in diabetes patients with stiff shoulders and RCTs. In addition, controversy exists regarding global or partial capsular release for stiff shoulders. A recently published meta-analysis also reported no additional benefit from global capsular release compared with partial release [49]. Several studies have reported that MUA combined with capsular release can reduce the complications of capsular release, although the complication rates of both MUA and capsular release were low, at 0.4% and 0.6%, respectively [3]. In addition, a recent randomized clinical trial also confirmed that the addition of capsular release during ARCR reduces the incidence of postoperative stiffness without affecting the postoperative outcome [50]. The comparison of different release methods might be a focus for future high-quality studies.

## Limitations

Several limitations exist in this study. First, all included studies were level II or III studies. However, level II-III studies or worse are commonplace in the orthopedic literature, indicating that this is the highest level of evidence we can obtain now. Second, several demographic factors related to a higher rotator cuff retear rate may also be potential confounders in this study. Relevant data include age, tear size, duration of symptoms and incidence of diabetes mellitus [44]. In this study, the stiff group had more patients with diabetes mellitus, which is consistent with the epidemiology of stiff shoulder. The effectiveness of surgical intervention for diabetes patients with stiff shoulder or rotator cuff tears has been proved [51–53]. Moreover, the results of this study did not yield higher retear rate in the stiffness group, which may enhance the strength of our conclusion. Third, a clear definition of a stiff shoulder was lacking and the included studies had different definitions, which may affect the accuracy of group division. However, a stiff shoulder describes a patient who presents with restricted ROM defined by the Upper Extremity Committee of ISAKOS [2]. Given that restricted ROM was confirmed under anesthesia in all studies and the significant ROM differences between groups, the impact of unclear definitions on the results may be insignificant. Last, not all studies used similar rehabilitation protocols, which may have influenced the results. Considering that most of the included studies used accelerated postoperative rehabilitation protocols and the support from existing studies, we recommend that all patients after one-stage treatment use the patient-based accelerated postoperative rehabilitation protocol for better outcomes.

## Conclusions

One-stage treatment for RCTs in stiff shoulders provides comparable ROM and patient-reported clinical outcomes as RCR for non-stiff RCTs. In addition, the rate of postoperative retear in stiff shoulder treated with one-stage treatment was not higher than in non-stiff shoulders. Future high-quality studies should focus on optimal one-stage treatment and protective factors for a lower postoperative retear rate. The comparable results found in this study can provide a reference for surgeons using early surgical repair for patients with coexisting RCTs and shoulder stiffness without the burden of the fear of undesirable results.

## Data Availability

The datasets used during the current study are available from the corresponding author on reasonable request.

## Abbreviations

RCTs: Rotator cuff tears
ROM: Range of motion
MUA: Manipulation under anesthesia
ARCR: Arthroscopic rotator cuff repair
FF: Forward flexion
ER: External rotation
IR: Internal rotation
ASES: American Shoulder and Elbow Surgeons
UCLA: University of California Los Angeles Shoulder Score
SST: Simple Shoulder Test
VAS: Visual analog scale
